# Visual Internal Urethrotomy for Adult Male Urethral Stricture Has Poor Long-Term Results

**DOI:** 10.1155/2015/656459

**Published:** 2015-10-01

**Authors:** Waleed Al Taweel, Raouf Seyam

**Affiliations:** ^1^Department of Urology, King Faisal Hospital and Research Center, Riyadh 11211, Saudi Arabia; ^2^Faculty of Medicine, Suez Canal University, Ismailia, Egypt

## Abstract

*Objective*. To determine the long-term stricture-free rate after visual internal urethrotomy following initial and follow-up urethrotomies. *Methods*. The records of all male patients who underwent direct visual internal urethrotomy for urethral stricture disease in our hospital between July 2004 and May 2012 were reviewed. The Kaplan-Meier method was used to analyze stricture-free probability after the first, second, third, fourth, and fifth urethrotomies. *Results*. A total of 301 patients were included. The overall stricture-free rate at the 36-month follow-up was 8.3% with a median time to recurrence of 10 months (95% CI of 9.5 to 10.5, range: 2–36). The stricture-free rate after one urethrotomy was 12.1% with a median time to recurrence of eight months (95% CI of 7.1–8.9). After the second urethrotomy, the stricture-free rate was 7.9% with a median time to recurrence of 10 months (95% CI of 9.3 to 10.6). After the third to fifth procedures, the stricture-free rate was 0%. There was no significant difference in the stricture-free rate between single and multiple procedures. *Conclusion*. The long-term stricture-free rate of visual internal urethrotomy is modest even after a single procedure.

## 1. Introduction

Male urethral stricture continues to be a common and challenging urologic condition. Despite the high failure rate of visual internal urethrotomy (VIU), it remains the most commonly performed procedure for the treatment of urethral strictures [1–7]. Even when VIU is initially performed selectively for short bulbar strictures under optimal conditions, the recurrence rate at 12 months was approximately 40% for strictures shorter than 2 cm. VIU and/or urethral dilation is usually the initial treatment approach offered in most cases of male urethral stricture, with no difference in efficacy between urethral dilation and urethrotomy [8–10]. Repeated urethrotomies were not associated with an improved success rate, and VIU for longer strictures usually failed [11, 12]. Urethral reconstruction is usually offered only after repeated failed transurethral stricture treatments, which in some cases span several years [13]. Unfortunately, repeated transurethral manipulation of bulbar strictures is associated with increased stricture complexity, stricture length, and a marked delay to curative urethroplasty [14]. Few studies have shown long-term follow-up of patients after VIU [11].

The purposes of this study are to report the overall success rate of VIU and to analyze whether repeated VIUs are associated with a long-term stricture-free rate. This study reflects urologic practice in real-life situations by multiple urologists in a busy tertiary care hospital.

## 2. Materials and Methods

This is a retrospective study of male patients who presented to the Department of Urology and underwent VIU for urethral stricture disease between July 2004 and May 2012. We evaluated the long-term stricture-free rate after visual internal urethrotomy following initial and subsequent urethrotomies.

We extracted data from medical records and our Integrated Clinical Information System on ascending urethrogram findings, including the site and length of stricture, number of previous urethrotomies, and presence of complex stricture (after urethroplasty or after radiation). All patients with symptoms or signs suggestive of urethral stricture underwent a urethrogram to confirm the diagnosis and determine urethral stricture length. All patients underwent cystourethroscopy before urethrotomy, confirming the diagnosis.

Four urologists performed the urethrotomies using a single incision at the 12 o'clock position or using a modified procedure including multiple radial incisions at the 3, 9, and 12 o'clock positions; the incisions were made with a cold knife or laser. Associated fossa navicularis stricture was treated with meatotomy prior to urethrotomy. Penile urethral strictures were treated with cold knife urethrotomy.

Follow-up data included subjective and objective results and whether subsequent intervention was needed. Symptoms of recurrence included decreased force of the urine stream, feelings of incomplete bladder emptying, or recurrent urinary tract infections. Signs of recurrence were a significant increase in postvoid residual urine on bladder ultrasound or bladder scan, decreased urine flow rate (<15 mL/second), or stricture as determined by diagnostic cystoscopy or retrograde urethrogram. Absence of symptoms or signs of recurrent stricture in any patient at last follow-up defined the success of the procedure. The end point of the follow-up was the last visit that showed failure of treatment or being recurrence-free for 36 months. Only data up to the fifth recurrence after repeated urethrotomy were included.

The Kaplan-Meier method was used to evaluate the stricture-free rate (survival function) after the first, second, third, fourth, and fifth urethrotomies. We used the Statistical Package of Social Science (SPSS, version 20, IBM Corporation, NY, USA). The log-rank test was used to compare survival differences between procedures.

## 3. Results and Discussion

### 3.1. Results

The mean age was 37 years (range: 17–82). A total of 446 male patients with urethral stricture disease were identified in the computerized records of the Department of Urology. Sixty-three patients were lost during follow-up. We excluded 82 patients who had complex urethral strictures, strictures longer than 5 cm, or dense palpable spongiofibrosis. This left 301 eligible patients who continued follow-up until the failure of urethrotomy was observed, at which point an alternative management plan was offered to them. We reported the duration of follow-up and time to failure of urethrotomy as the same duration. Further management and follow-up are excluded from this paper.

The stricture characteristics are shown in Table 1. The most common location is bulbar urethral stricture in 227 (75%) patients, penile urethral stricture in 36 (11%) patients, combined penile and bulbar urethral stricture in 24 (8%) patients, and fossa navicularis stricture in 14 (5%) patients. The mean stricture length was 13 mm (range: 4–42). The overall stricture-free rate at the 36-month follow-up was 8.3% with a median time to recurrence of 10 months (95% CI 9.5 to 10.5, range: 2–36). The success rate following single urethrotomy was modest and dropped significantly after repeated urethrotomies (Table 2).

**Figure 1:**
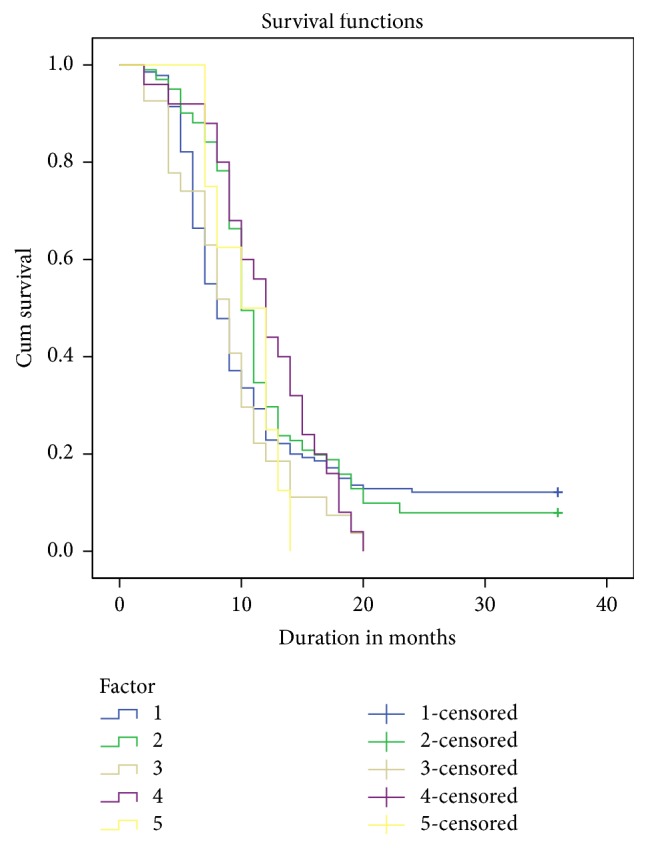
Stricture-free probability after the first, second, third, fourth, and fifth urethrotomies (Kaplan-Meier survival analysis).

Most recurrences occurred within the first postoperative year. Survivors or patients without recurrence were only those with a stricture length of <1 cm and in the bulbar urethra. There was no significant difference in the survival analysis of duration to recurrence among patients undergoing single or multiple procedures (*p* = 0.181, Figure 1). There was no significant difference in the outcome based on the length of the stricture or the type of treatment.

### 3.2. Discussion

Urethral strictures are often treated with urethrotomy, most commonly direct visual internal urethrotomy [15]. With the introduction of lasers, holmium laser urethrotomy was subsequently used in many centers with equal recurrence outcomes as achieved with VIU [16, 17]. Many urologists prefer VIU over urethral reconstruction because of its ease to perform, low cost, short hospital stay, and perceived low complication rate. They may opt to repeat VIU several times to avoid complex urethral reconstruction, which requires significant surgical experience. This trend continues despite the moderate success rate reported in the selected patients. To reduce the stricture recurrence rate, several investigators evaluated different intralesional adjuvant injections with variable success [18–23]. We set out to report the results of VIU of our patients, including a wider inclusion base and strict criteria of success in a long follow-up period. We felt that these patients constitute a real patient group that tempts urologist to repeatedly administer VIU for the management of their stricture.

Our stricture-free rate of 8.3% at a median of 10 months (range: 2–36) is much lower than that reported by others on long-term follow-up [24]. Heyns et al. found that, after a single dilation or urethrotomy in patients who did not experience restricture within 3 months, the estimated stricture-free rate was 50–60% at 48 months [24]. The higher success rate in that study might be related to the exclusion of patients who failed the treatment in the first three months from the analysis and the shorter stricture length. Another study reported a 32% recurrence-free rate after a median follow-up of 98 months following a single internal urethrotomy. The prognostic characteristics of bulbar urethral strictures associated with good results included single or primary strictures and length shorter than 10 mm [11, 25]. The inclusion of strictures from 1 to 4 cm and the strict success criteria in our study might explain a more realistic success rate of 12.1% after single VIU. Comparison of studies that evaluate the outcome of stricture urethra treatment is greatly affected by the success criteria. This heterogeneity of the definition of success has been clearly shown in a meta-analysis of urethroplasty outcome involving more than 300 articles [26]. We did not separately report the details of the differences in outcome between different stricture lengths, associated location, or type of treatment because there was no significant difference. A focus on these comparisons would have been extremely relevant if we had a significant success rate. However, the overall success rate was poor. Only 25 patients remained stricture-free at 10 months. Compared to the total of 301 patients, subgroup analysis did not show a significant difference because of the small number of successful cases in each comparison cell.

Repeated VIU was associated with more dismal outcomes. This is in accordance with the previously reported data [11, 24, 27]. We found no significant advantage of single versus repeated VIU. We think that the inclusion of long strictures at different sites masks the claimed advantage of single VIU. Our findings stress that an early attempt at urethroplasty is warranted. This is particularly important because repeated urethrotomies have a negative impact on the success of subsequent urethroplasty [28].

Several studies have examined the cost-effectiveness of managing anterior urethral strictures. Urethroplasty as the primary therapy was cost-effective only when the expected success rate of the first VIU was less than 35% [29], whereas VIU became more favorable when the long-term risk of stricture recurrence was less than 60% [30]. If a repeat urethrotomy is required, open urethroplasty is the treatment of choice for recurrent urethral stricture.

## 4. Conclusions

Visual internal urethrotomy is a simple and popular treatment for male urethral stricture; however, the long-term stricture-free rate is modest even after only a single procedure. Most of the recurrences were found to occur within one year. Thus, definitive curative reconstruction should be planned as early as possible. Repeated visual internal urethrotomies should be considered only in patients who are poor surgical candidates and not because of the convenience of performing a simple procedure.

## Figures and Tables

**Table 1:** Stricture characteristics.

Stricture length	Location	Number of patients
<1 cm	Penile Bulbar Penile and bulbar Fossa navicularis	14 75 0 2

1-2 cm	Penile Bulbar Penile and bulbar Fossa navicularis	16 87 10 4

>2 cm	Penile Bulbar Penile and bulbar Fossa navicularis	6 65 14 8

**Table 2:** Urethrotomy and stricture-free rate.

Number of urethrotomies	Stricture-free rate	Median time to failure (months)	Number of stricture-free patients	Total number of patients (%)
First	12.1%	8 (95% CI 7.1 to 8.9)	17	140 (46.5%)
Second	7.9%	10 (95% CI 9.4 to 10.6)	8	101 (33.6%)
Third	0%	9 (95% CI 7.3 to 10.7)	0	27 (9%)
Fourth	0%	12 (95% CI 10.4 to 13.6)	0	25 (8.3%)
Fifth	0%	10 (95% CI 6.3 to 13.7)	0	9 (3%)
Overall	8.3%	10 (95% CI 9.5 to 10.5)	25	301 (100%)
